# Kratopenia as an indicator of sarcopenia in smokers: Cut-off points for peak knee torque

**DOI:** 10.1371/journal.pgph.0004495

**Published:** 2025-04-24

**Authors:** Paolla Sanches, Karina A. de S. Souza, Alexandre R. P. Ambrozin, André R. Pereira, Leandro L. da Silva, Amanda S. Cano, Luciana Pinato, Tiago V. Barreira, Dionei Ramos, Mahara Proença

**Affiliations:** 1 Postgraduate Program in Human Development and Technologies, São Paulo State University (UNESP), Rio Claro, Brazil; 2 Postgraduate Program in Human Movement Sciences, State University of Northern Paraná (UENP), Jacarezinho, Brazil; 3 Postgraduate Program in Physiotherapy, São Paulo State University (UNESP), Presidente Prudente, Brazil; 4 Department of Physiotherapy and Occupational Therapy, São Paulo State University (UNESP), Marília, Brazil; 5 Postgraduate Program in Speech, Language and Hearing Sciences, São Paulo State University (UNESP), São Paulo, Brazil; 6 Department of Speech, Language and Hearing Sciences, São Paulo State University (UNESP), Marília, Brazil; 7 Falk College, Exercise Science Department, Syracuse University, Syracuse, New York, United States of America; PLOS: Public Library of Science, UNITED STATES OF AMERICA

## Abstract

Muscle strength is a crucial predictor of adverse outcomes and is essential for identifying kratopenia and physical limitations. Smoking can aggravate this condition, damaging the musculoskeletal system. Assessing muscle strength, especially with portable dynamometers, is essential for early detection of muscle dysfunction. Studies demonstrate the importance of standardizing protocols and defining cut-off points for peripheral muscle weakness in general. Thus, focusing on the effects of smoking on muscle function, the objective was to investigate cut-off points based on functional limitation and identify the presence of kratopenia in smokers. This cross-sectional study, composed of smokers (conventional cigarettes), regardless of gender, aged over 18 years, was conducted with a comprehensive approach. The volunteers were evaluated by personal data, carbon monoxide analysis (monoximetry), and physical-functional aspects such as lung function (spirometry), functional performance (6-minute walk test, sit-to-stand, fourmeter gate speed, and physical activity in daily life), and peripheral muscle strength (portable digital dynamometry). A total of 143 smokers were evaluated, with high levels of tobacco dependence and preserved lung function. Men showed significantly higher peripheral muscle strength across all variables (p < 0.05). ROC analysis revealed acceptable discrimination for detecting kratopenia from functional limitation: AUC of 70% for knee extension (p < 0.01) from the cut-off points identified for knee extension lower than 214.8Nw and 273.6Nw; and 70% for knee flexion (p < 0.01) lower than 125 Nw and 156.2Nw, women and men, respectively, to be loss of muscle strength and power. Kratopenia was present in > 50% of participants based on peak torque (Nw) (56% of knee extension and 52% of knee flexion), being more prevalent in women. Peak knee torque measurements, especially those of extension and flexion, can determine kratopenia in limited functional smokers. Determining specific cut-off points offers an effective tool to identify and prevent sarcopenia risk in smokers.

## Introduction

Sarcopenia is a modification of skeletal muscle characterized by progressive and generalized loss of muscle mass and strength and reduced functional physical performance [1,2]. Muscle strength precedes muscle mass as the best predictor of adverse outcomes, and it plays a crucial role in health [3]. It’s not just a number on a scale, but a powerful tool that can help identify sarcopenia risk [4]. The earlier a muscle dysfunction diagnosis is made, the greater the chances of effective rehabilitation.

Particularly, the kratopenia [4] term refers to the isolated loss of muscle strength and power, which can occur without a notable reduction in muscle mass. These conditions are clinically important as it can indicate an increased risk of developing sarcopenia [2–5]. Therefore, measuring muscle strength is essential for identifying the onset of functional loss [4,5].

Lifestyle behaviors, such as smoking, may contribute to the development of kratopenia [4]. Exposure to cigarette smoke has harmful effects on the musculoskeletal system, diminishing contractile tension and inhibiting protein production. This is due to cardiovascular and metabolic problems that are worsened by oxidative stress in the musculoskeletal system induced by smoking [6–10]. Therefore, examining the negative impact of smoking on muscle strength is essential for health.

There are several instruments available to assess muscle strength, including isokinetic devices and handheld digital dynamometers, which measure different muscle contractions [11]. One particularly reliable method is measuring the peak knee torque with a dynamometer, as it provides an objective and quantitative assessment of strenght deficiencies associated with sarcopenia [12–14]. Portable dynamometry is increasingly accessible in various clinical settings, facilitating the early detection of muscle dysfunction.

Ramos et al. [15] demonstrated that portable dynamometry can effectively detect muscle mass loss in individuals with Chronic Obstructive Pulmonary Disease (COPD). Their findings indicated that participants with reduced knee extension strength were 5.9 times more likely to experience this mass loss, regardless of sex, disease stage and functional capacity [15]. Additionally, Garcia and collaborators also used the same instrument to assess the strength and determine the prevalence of muscle weakness in participants with idiopathic disease. Their study revealed that between 20% to 46% of these patients had a generalized peripheral weakness, varying by muscle group, with a particularly high incidence of reduced strength in the lower limbs [16]. These studies highlight the importance of standardizing protocols and establishing cut-off points for assessing muscle weakness in specific population, which can help to prevent sarcopenia.

Given the detrimental effects of smoking on muscle function, this study aims to establish cut-off points for knee muscle strength in smokers. Defining these thresholds will help detect physical disability and characterize the presence of kratopenia in this population, enabling early intervention strategies. The specific research question addressed is: What are the cut-off points for knee muscle strength in smokers as determined by portable digital dynamometry? This research has the potential to significantly advance the field of muscle strength assessment and its implications for specific populations.

## Materials and methods

### Ethics statement

Ethical approval for the study was granted by the Research Ethics Committee of the São Paulo State University (UNESP) Faculty of Sciences and Technology, Presidente Prudente - SP, Brazil (Approval No. 3,424,962). Participants were informed about the objectives and procedures of the study and agreed to participate by signing a written informed consent form (ICF) by the Declaration of Helsinki.

### Study design

This cross-sectional observational study followed the Reporting of Observational Studies in Epidemiology (STROBE) [17] guidelines for reporting observational studies Data Collection from 08/07/2019–10/09/2020.

### Participants

The study included smokers of both genders aged 18 to a maximum of 60 years. Participants were recruited through local media and had to smoke at least five cigarettes per day [18]. Exclusion criteria included any pathological conditions that could interfere with testing, as well as individuals who incomplete strength assessment because of discomfort, pain, or had difficulty understanding the tests. Ex-smokers or other types of cigarettes were also not included.

### Assessments

To perform the assessments, participants were required to avoid alcohol, caffeine, bronchodilator medications, and moderate-to-vigorous physical activity for 12 hours prior to the evaluation. They also abstained from smoking for 12 hours, ensuring a cut-off of ≤ 10 ppm [19] for exhaled carbon monoxide measurement using a micro CO monitor (Micro Medical Ltd., Rochester, Kent, UK). Initially, participants underwent an interview to gather personal identification data, anthropometric measurements (such as body mass index (BMI)), smoking history (including cigarettes/day and years of smoking) [20], nicotine dependence (using the Fagerstrom Test for Nicotine Dependence Dependence) [21], self-reported comorbidities. Following this, they underwent lung function tests, assessment of physical-functional performance and peripheral muscle strength.

#### Lung function tests.

In accordance with the guidelines set by the European Respiratory Society and American Thoracic Society, the pulmonary function tests were performed using spirometry [22]. A portable spirometer (Spirobank-Mir, Italy) was utilized to measure Forced Expiratory Volume in 1 sec (FEV_1_), Forced Vital Capacity (FVC), and FEV_1_/FVC ratio, using reference values to the Brazilian population [23,24].

#### Physical-functional performance assessment.

Sarcopenia is a complex condition that requires assessment through multiple functional tests, meaning a comprehensive battery of physical performance evaluations is necessary [14]. In this study, we propose to calculate the overall score by summing the results form the following four tests: the 6-minute walk test (6MWT) [25,26], Four-meter gait speed (4MGS) [27], five repetitions sit-to-stand *(5rep-STS)* [27], and physical activity level in daily life [28]. Functional limitation is determined by the total scores from these physical performance tests, classifying participants into two categories: regular and limited. Specifically, participants with a combined score of the four functional tests of 2 points or higher are classified as limited (1), while those with a score of 1 point or lower are classified as regular (0).

***6-minute walk test****:* Each participant performed the 6MWT in accordance with the guidelines established by the American Thoracic Society. The results were analyzed using the normative reference values suggested by Soares (2011) [25]. Values equal to or below 80% of the predicted distance covered in the test were classified as “lower”, and values above the predicted distance were classified as “normal” (0 = normal or 1 = lower).

***Four-meter gait speed***: The 4MGS area was marked with cones, and the participant was instructed to walk normally, as if they were walking on the street. The individual had to cross to the other side, and the duration was set [27]. Those who took 5.1 seconds or longer to complete the test were classified as “lower”, while those who took less time were classified as “normal”. The classifications were denoted as follows: 0 = normal, 1 = lower.

***Sit-to-stand***: For the 5rep-STS, the participant was positioned in a standard-height chair with their arms crossed over their chest and their feet flat on the floor. They were asked to stand up. If a participant was unable to stand, the test was terminated. If they were able to stand, they were instructed to complete five consecutive repetitions, timing how long it took them to complete it [27]. Participants who took 12 seconds or longer to complete the test were classified as “lower” ability, while those who finished in less time were classified as “normal” (0 = normal, 1 = lower).

***Physical activity in daily life (PADL)***: Physical activity was monitored through pedometry, using pedometers (YAMAX PW-611- Power Walker Pedometer, USA, Inc., San Antonio, TX, USA) to record the total number of daily steps [28]. Participants wore the device for seven consecutive days, only removing it during bathing and sleeping. At the end of each day, the total number was recorded. The average number of steps per day was calculated to assess habitual physical activity. Individuals who took more than 7.500 steps per day were categorized as active, while those 7.500 steps or fewer were categorized as inactive [28] (0 = active or 1 = inactive).

#### Kratopenia assessment - peripheral muscle strength.

Peripheral muscle strength was assessed using a portable digital dynamometer (SKILL-TEC – Model SKDD-100), and all measurements were conducted in the morning, from 8 AM to 1 PM, during a single day of assessment. The focus was on evaluating two muscle groups: the quadriceps (knee extension) and the hamstrings (knee flexion). A portable dynamometer [15] was securely attached to a specially designed chair for the test and connected to the device using a load cell to ensure standardization.

To evaluate the quadriceps, the participant was seated in a chair with an upright posture, utilizing back support. Their hips and knees were flexed at 90 degrees, and their hands rested on their thighs. The participant performed a knee extension movement. As for the hamstrings, the participant remained in the same seated position with upright posture, back support, hips and knees flexed at 90 degrees, and hands on their thighs. In this case, the movement performed was knee flexion.

Muscle strength was evaluated using maximal voluntary isometric contractions (MVIC). Each contraction lasted six seconds, and participants were allowed to perform up to ten repetitions for each muscle group. The maximal voluntary contraction was determined by taking the best result from three reproducible trials, which reflected the maximum force sustained for one full second. A minimum of four and a maximum of fifteen measurements were conducted for each muscle group. The highest MVIC value for each group was included in the analysis, provided that the two highest values differed by no more than 5%.

Participants performed maximal voluntary isometric contractions with one-minute intervals between attempts. Peak force and mean force values were recorded in Newtons (N) and kilogram-force meters (kgfm). A single, experienced evaluator trained in isometric dynamometry conducted the tests, using standardized verbal commands to enhance the reproducibility of the results and ensure the reliability of measurements taken by the same evaluator. The dynamometer was calibrated annually.

### Statistical analysis

Statistical analysis was performed using the Statistical Package of Social Science 22.0 software (SPSS Inc., Chicago, IL, USA). Initially, the data were described using measures of central tendency and dispersion. Due to non-normal distribution of the data, variables were expressed as medians with interquartile range (25–75%). Categorical variables were expressed as absolute and relative frequencies. The Mann-Whitney test, suitable for non-parametric data analysis, was used to compare lung and peripheral muscle function variables between genders.To determine the probability of detecting a true effect, the power of the study was calculated using G-Power 3.1 software. For the Mann-Whitney U test analysis, the estimated power (r) [29] was 58% for extension (r = 0.32) and 71% for flexion (r = 0.38), with an alpha error probability of 0.05. The simple size 1 consisted of 76 females and simple size 2 of 65 males.

Currently, there are no established cut-off points in the literature regarding the peripheral muscle strength of smokers, specifically for the quadriceps and hamstrings. To address this gap, we opted to use percentiles (10th, 25th, 50th, 75th, 90th, and 95th) from the strength distribution as a methodological strategy. This approach enables us to identify potential values that reflect different levels of muscular performance. It allows for the exploration of possible classifications of weakness, facilitating both exploratory and comparative analyses. Additionally, this method has several advantages: it uses data from the study sample itself, accurately reflecting the variation of the variable within that sample; it identifies clinical extremes (low or high percentiles may indicate frailty or good performance); and it offers flexibility for future analyses, as percentiles can be utilized to generate hypotheses and validate them in subsequent studies.

To assess the sensitivity and specificity of knee extension and flexion measurements obtained from a dynamometer, we employed Receiver Operating Characteristic (ROC) curve analysis. Functional limitation was defined as the total score from each physical-functional performance test, resulting in a dichotomized outcome (0 = normal or 1 = limited). ROC analysis was conducted to evaluate the accuracy of peak torque in detecting kratopenia [30], with functional limitation as the dependent variable and peak torque (measured in Newtons) as the independent variable. A total of 64 participants were included in the analysis, comprised of 26 in the normal group and 38 in the limited. Therefore, the suggested cut-off points for peak torque measured in Newtons were determined based on the Area Under the Curve (AUC), which aims to maximize sensitivity and specificity. AUC values of 0.70 or higher were considered to indicate acceptable discriminative capacity, based on statistical criteria established for diagnostic tests, as suggested by Okeh and Okoro (2012) [31]. We selected two areas that exhibited the best sensitivity and specificity for both extension and flexion. The significance level for all statistical tests was set at p < 0.05.

## Results

### Characteristics of participants

**Fig 1:**
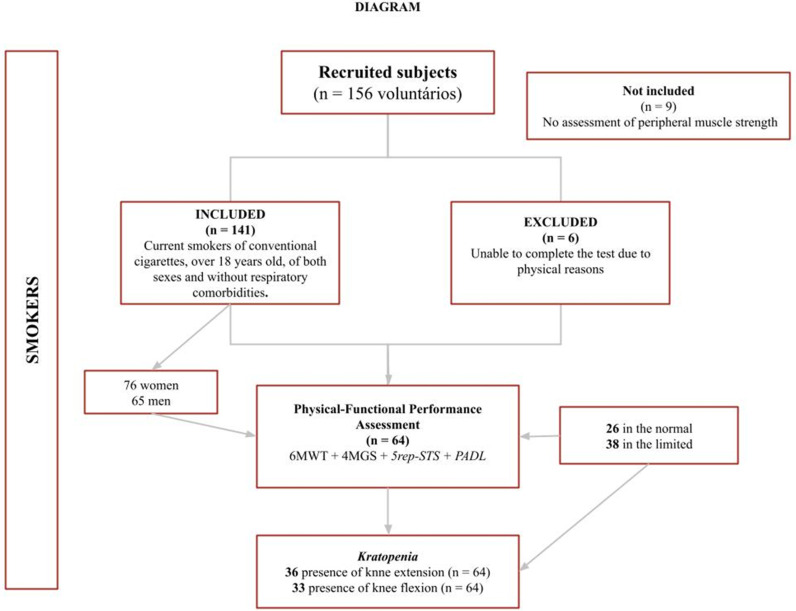
Flowchart of participant recruitment and assessment. Flowchart illustrates the recruitment and assessment process of smokers included and excluded in the study.

A total of 156 participants (supplementary material) were recruited for the study. Six smokers were excluded, and an additional nine were not included (as shown in the diagram, see Fig 1).

Thus, 141 smokers (54% women) without a prior diagnosis of lung disease were considered for the analysis of general physical health and function (see Table 1).

**Table 1:** General characteristics of the sample.

	Overall (N = 141)	Women (N = 76)	Men (N = 65)
**Anthropometric**			
Age. years	41 [32 – 49]	43 [33 – 52]	39 [30 – 48]
BMI. kg/m^2^	26 [22 – 29]	26 [22 – 29]	25 [23 – 30]
**Smoking status**			
CigarettesXday. amount	20 [15 – 20]	20 [10 – 20]	20 [15 – 30]
Time to smoke. years	20 [14 – 30]	20 [13 – 32]	20 [14 – 30]
YearsXpack. amount	10.4 [5.6 – 18.0]	7.74 [5.46 – 15.21]	12.81 [6.82 – 19.90]
Fagerstrom. points	6 [5 – 7]	6 [5 – 7]	6 [4 – 7]
**Lung function**			
%FVC	89 [83 – 99]	88 [79 – 99]	90 [85 – 100]
%FEV_1_	90 [82 – 97]	90 [80 – 99]	91 [82 – 97]
FEV_1_ /FEC	90 [82 – 100]	88 [81 – 96]	92 [82 – 100]
%FEF_25–75%_	87 [70 – 106]	89 [70 – 114]	87 [72 – 102]
**Physical-Functional Performance**			
**6MWT**			
Walking distance (DP). meters	565 [519 – 613]	554 [511 – 577]	585 [545 – 637]^*^
% predito da DP	87 [80 – 94]	85 [81 – 92]	90 [82 – 96]^*^
**PADL**			
Steps per day	7.047 [5.067 – 9.135]	6.362 [4.662 – 8.728]	7.115 [5.554 – 9.757]
**4MGS**			
4m. score	4 [3 – 4]	4 [3 – 4]	4 [3 – 4]
4m. time	4.49 [3.97 – 5.05]	4.49 [3.97 – 5.20]	4.47 [3.95 – 4.96]
**5rep-STS**			
STS. score	2 [1 – 3]	2 [1 – 3]	2 [1 – 3]
STS. time	14.86 [11.96 – 16.93]	14.91 [11.75 – 16.27]	13.74 [12.10 – 17.36]

**Note.** Data presented as median [interquartile range].

*statistical significance *p* ≤ 0.05 (Mann-Whitney) between sexes with small size power.

Table 1 present’s descriptive statistics related to anthropometric, body composition, and performance variables. In terms of general physical-functional aspects, smokers predominantly presented high levels of tobacco consumption and dependence, while their lung function remained within predicted values. Regarding physical performance, smokers showed physical inactivity and low results in the sit-to-stand test, although their gait speed was still considered adequate. A statistically significant difference was observed between the sexes in terms of distance covered in meters (SD) (*U* = 1,207, *z* = -5.402, *p* < 0.001) and percentage of predicted (%prev) for the 6MWT (*U* = 2,032, *z* = -2.061, *p* < 0.001). However, both differences had small effect sizes (0.45 and 0.29 respectively).

In general (n = 141), peripheral muscle strength was measured in newtons (N). The median knee extension strength was found to be 23.9 [17.1–30.3] kgfm, which is equivalent to 234.8 [168.4–297.0] N. Additionally, the median strength for knee flexion was 13.0 [9.2–17.2] kgfm, corresponding to 128.4 [91.6–169.2] N.

Significant differences between the sexes revealed a notable disparity in muscle strength, with men exhibiting higher values in all muscle strength variables. The women participants showed a knee extension strength of 20.6 [15.8–26.2] kgfm, or 199.8 [155.8–257.8] N, whereas men demonstrated significantly higher values of 27.7 [21.7–32.8] kgfm, or 272.4 [213.8–322.3] N (*U* = 1,596, *z* = -3.82, *p* < 0.05). However, the effect size was low (r = 0.32). For knee flexion strength, women presented a value of 11.0 [7.9–14.6] kgfm, or 111.2 [80.1–144.8] N, while men displayed higher values of 16.0 [11.6–19.7] kgfm, corresponding to 157.4 [114.5 – 193.9] N (*U* = 1,431, *z* = -4.49, p < 0.05), also with low effect size (r = 0.38).

### Cut-off points for peak knee torque from physical-functional performance

The strength for knee extension and flexion (n = 64) was categorized according to seven percentiles (≤10º. ≤ 25º. ≤ 50º. ≤ 75º. ≤ 90º. ≤ 95º) to establish suggested cut-off points for the muscle groups (Table 2).

**Table 2:** Muscle strength values based on percentiles.

Move	N = 64					
	≤10	≤25	≤50	≤75	≤90	≤95
**Knee Extension (N)**						
Woman	134.0	168.4	214.8	290.8	320.6	408.4
Men	158.4	210.2	273.6	343.1	441.0	500.0
**Knee Flexion (N)**						
Woman	67.6	87.8	125.0	149	177.0	199.7
Men	67.4	109.1	156.2	183.7	242.4	259.8
**Note.** N: newton.						

**Fig 2:**
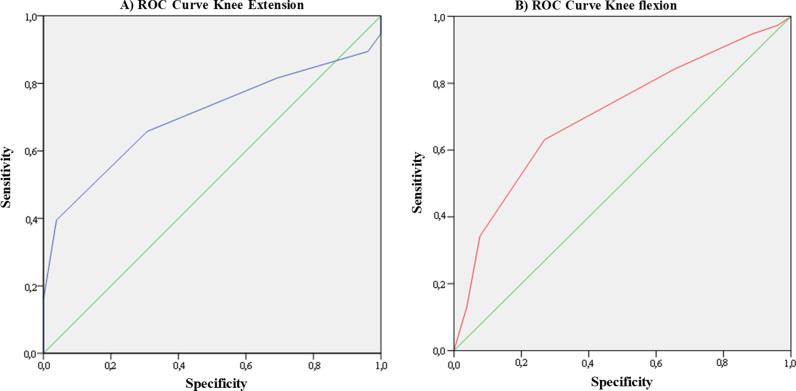
ROC curve of peripheral muscle strength based on maximizing sensitivity and specificity. (A) The cut-off points for peak torque, measured in Newtons, are provided for knee extension, based on the two areas with the best sensitivity and specificity. (B) The cut-off points for peak torque, measured in Newtons, in knee flexion are also derived from the two areas with the best sensitivity and specificity.

ROC analysis was performed to assess the accuracy of peak torque measurements. For knee extension, the ROC results demonstrated a statistically significant curve (AUC = 0.701, Standard Error = 0.065; p < 0.01; CI = 0.574 - 0.829). This suggests that, in a random selection, 70% (CI 0.574 - 0.829) of cases with performance limitation will present higher scores than those without limitation, indicating acceptable discrimination in predicting mobility limitation (see Fig 2A). Similarly, for knee flexion, the ROC results also showed a statistically significant curve (AUC = 0.707, Standard Error = 0.065; p < 0.01; CI = 0.579 - 0.835). This indicates that 70% (CI 0.579 - 0.835) of cases with limitation will score higher than those without limitation, confirming an acceptable discrimination (see Fig 2B).

Strength can vary between sexes, which led to the establishment of cut-off points for both women and men. For knee extension, the cut-off points were identified as ranging from 214.8 to 290.8 N for women and from 273.6 to 343.1 for men. This resulted in a sensitivity of 0.81 and a specificity of 0.69. For knee flexion, the cut-off points were between 125.0 to 149.0 N for women and 156.2 to 183.7 for men, yielding a sensitivity of 0.84 and a specificity of 0.73.

These cut-off points indicate that participants within these ranges exhibit normal strength, while lower values suggest the presence of kratopenia among smokers in this study. Specifically, 56% of smokers in this sample presented with kratopenia based on peak torque (Nw) of knee extension, with 33% of women and 23% of men affected. In terms of knee flexion, 52% of individuals were affected, comprising 31% of women and 22% of men.

## Discussion

The identified cut-off points for quadriceps and hamstring strength can serve as practical screening tools to identify smokers who are at an increased risk of sarcopenia, a condition that can limit mobility and functional performance. Due to the progressive nature of muscle weakness in smokers, early identification using their thresholds allows healthcare professionals - such as physiotherapists and rehabilitation specialists - to implement targeted interventions before severe functional decline occurs. Smokers with muscle strength below the 10th or even 25th percentile may benefit from structured resistance training programs, protein supplementation strategies, or, if appropriate, neuromuscular electrical stimulation.

Smoking is a leading cause of both mortality and morbidity, and it is recognized as a significant risk factor for the onset of numerous diseases. Musculoskeletal health is an area where we can investigate the changes caused by smoking. Smoking exposes the body to chemicals present in oxygen contaminated by smoke, leading to harmful effects not only on the pulmonary but also on the extrapulmonary systems [10,11,32]. Smoking addiction can contribute to the development of kratopenia, a critical risk factor for sarcopenia, through several mechanisms [4]. These include chronic inflammation, oxidative stress [9], mitochondrial dysfunction, and changes in muscle morphology [8–10,33]. Such biological processes can lead to accelerated degradation of muscle tissue, impairing the muscle’s ability to regenerate and worsening the decline in strength (kratopenia) and mobility. Our results align with these findings, indicating that kratopenia can occur in smokers.

This study is the first to propose cut-off values for knee torque using a more accessible strength test. It established sex-specific cut-off values for knee extension: below 273.6 N for men and 214.8 N for women. For knee flexion strength, the cut-off values are below 156.2 N for men and 125.0 N for women. These values can now serve as references for screening for kratopenia in smokers. Previously, Ramos et al. [15] proposed cut-off points for muscle mass loss in chronic obstructive pulmonary disease (COPD) population, identifying values of 206.4 N for knee extension and 112 N for knee flexion in men, and 144.5 N for knee extension and 72.7 N for knee flexion in women. These findings are consistent with our results, which also indicate a decline in muscle strength primarily attributed to smoking habits linked to the development of COPD.

Additionally to the cut-off points, our results indicate that knee strength measurements using handheld dynamometry could be effective and cost-efficient alternatives for assessing muscle weakness, especially when compared to isokinetic equipment or more invasive methods. This approach is particularly useful for home assessments, resource-limited clinics, or rehabilitation settings. Futhermore, incorporating muscle strength screening into smoking cessation programs could enhance their effectiveness by addressing both pulmonary and musculoskeletal health.

While isokinetic testing provides highly controlled assessments at fixed speeds, portable dynamometry offers a more accessible, cost-effective alternative. However, it may be influenced by examiner technique and participant positioning. These methodological differences limit direct comparisons between studies and highlight the need for specific normative values for each assessment method. Future research should explore conversion factors between these methodologies or investigate the agreement between portable and isokinetic dynamometry in different clinical populations.

Our findings highlight the considerable effect of smoking on peripheral muscle strength and underline the need to establish specific cut-off points for this population. These values can serve as reference for future interventions aimed at recovering or maintaining muscle strength in smokers. Further research should explore whether improvements in muscle strength occur following smoking cessation and whether rehabilitation programs designed for smokers can help mitigate muscle deterioration over time.

### Limitations and future research

Despite the promising findings, our study has some limitations and areas for future research that should be considered. Firstly, our sample was restricted to smokers, which may limit the extrapolation of the results to other populations. Additionally, the cut-off points we established were based on the percentiles within this specific group, underlining the importance of validating these thresholds in diverse populations.

Another crucial point is that our study employed a cross-sectional design, meaning we cannot determine whether smoking cessation impacts muscle strength and its respective cut-off points over time. Future research should incorporate longitudinal studies to examine whether muscle strength can recover after quitting smoking and whether the proposed thresholds remain valid or need adjustment for individuals who successfully stop smoking. Such investigations would offer valuable insights into the reversibility of smoking-related muscle impairment and its implications for clinical and rehabilitation strategies.

## Conclusion

This study suggested that measuring knee torque values, particularly for extension and flexion, could help identify kratopenia in smokers with functional limitations, which is a key indicator of sarcopenia risk. Establishing specific cut-off points for knee strength may provide a valuable tool in clinical practice, potentially leading to improved muscle rehabilitation protocols before smoker’s progress to chronic lung disease. Additionally, this approach could serve as a strategy for encouraging smoking cessation.
